# Abducens Nerve Neuropraxia due to Acute Bacterial Rhinosinusitis: Case Report and Literature Review

**DOI:** 10.1155/2023/5175871

**Published:** 2023-11-27

**Authors:** Abdulrahman Alghulikah, Sarah Alseneidi, Hedayah Alsaady, Ahmed Alhussien, Surayie Al-Dousary, Saud Alromaih, Abdulrahman AlHumaizi

**Affiliations:** ^1^Otolaryngology–Head and Neck Surgery Unit, Surgery Department, Security Forces Hospital, Riyadh, Saudi Arabia; ^2^Children's Hospital, King Fahad Medical City, Riyadh, Saudi Arabia; ^3^Department of Surgery, Otolaryngology Division, Security Forces Hospital, Makkah, Saudi Arabia; ^4^Otolaryngology–Head and Neck Surgery Department, College of Medicine, King Saud University, Riyadh, Saudi Arabia; ^5^Department of Otolaryngology–Head and Neck Surgery, King Abdullah bin Abdulaziz University Hospital, Riyadh, Saudi Arabia

## Abstract

**Background:**

Acute bacterial rhinosinusitis (ABRS) is a common infection of the paranasal sinuses that can lead to complications such as orbital and intracranial extension. The abducens nerve course is adjacent to the sphenoid sinus. Diplopia is rarely the initial presentation of sphenoid sinus pathology. In this article, we present the case of a middle-aged male who presented with diplopia and abducens nerve palsy secondary to ABRS, and we conducted a literature review in search of similar cases. *Case Presentation*. A 52-year-old male presented with diplopia secondary to ABRS. Imaging revealed the complete opacification of the bilateral sphenoid and frontal sinuses, with the extension of the inflammatory process to the optic nerve and cavernous sinus. The patient underwent a surgical intervention, which revealed a pyocele collection in the opticocarotid recess inside the sphenoid sinuses. After the surgery, the patient received antibiotics and reported a complete recovery.

**Conclusions:**

Acute bacterial rhinosinusitis can present with atypical symptoms and lead to serious complications, such as abducens nerve palsy. Early diagnosis, appropriate management, and timely referral to a multidisciplinary team are crucial to preventing residual nerve damage and ensuring favorable outcomes.

## 1. Introduction

Acute viral rhinosinusitis triggers the activation of the immune cascade, which is responsible for common cold symptoms and helps to eradicate viral infection [1]. In chronic rhinosinusitis (CRS), the prolonged inflammatory reaction results in damage to the nasal epithelial cells' surfaces, as evidenced by altered protein expression [2]. In acute bacterial rhinosinusitis (ABRS), these changes in the cell architecture compromise the defense mechanism of the cell, increase susceptibility to infection, and create a favorable environment for bacterial invasion [3].

Acute bacterial rhinosinusitis is clinically defined as the presence of any three of these signs and symptoms: purulent discharge, severe headache, fever, the elevation of inflammatory markers such as the C-reactive protein/erythrocyte sedimentation rate, or double-interval illness [1]. However, a meta-analysis showed that it is difficult to distinguish viral from bacterial acute rhinosinusitis in clinical settings and that the prevalence of ABRS remains poorly defined [4].

The abducens nerve travels within the cavernous sinus, which makes it anatomically adjacent to the sphenoid sinus [5]. Diplopia is rarely the initial presentation of sphenoid sinus pathology. The abducens nerve can be affected by neoplasms, post-radiation changes, invasive fungal sinusitis, allergic fungal sinusitis, or bacterial sinusitis [6]. In this article, we present the case of a middle-aged male who presented with diplopia and abducens nerve palsy secondary to ABRS, and we conducted a literature review in search of similar cases.

## 2. Case Presentation

### 2.1. Clinical Presentation and Diagnosis

A 52-year-old male with a body mass index of 26.5 and a known case of bronchial asthma and CRS presented to the emergency department, complaining of double vision for one day, which improved with the closure of the right eye. He had also been experiencing a severe right frontal headache for three days, nasal discharge, and facial heaviness. He denied having any other visual complaints, a history of allergic symptoms, or nasal obstruction. The patient had two previous functional endoscopic sinus surgeries (FESSs), with the last having been completed in the tertiary center 18 months before presenting to the emergency department. Prior to his presentation, he had been using Mometasone nasal spray and normal saline nasal rinses for one month.

The ophthalmological examination demonstrated binocular double vision during the straight and right gaze at near distances, with right extraocular muscle limitation of around 20%, indicating a partial right-sided sixth-nerve palsy. Other ophthalmic examinations, including visual acuity, intraocular pressure, and slit lamp examinations, were unremarkable. Meanwhile, the endonasal scope showed grade 2 polyps on the right side, with purulent discharge from the middle meatus. The left side showed congested mucosa without polyps or purulent discharge. The remainder of the cranial nerve examination was normal. Laboratory tests were unremarkable.

**Figure 1:**
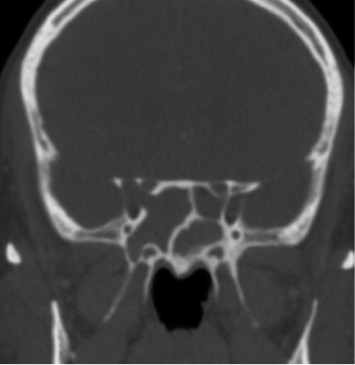
Computed tomography imaging showing diffuse opacification of the sinuses, with no skull base defect.

**Figure 2:**
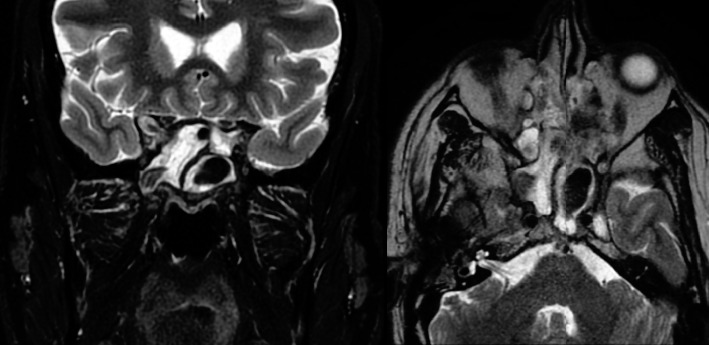
Coronal (left) and axial (right) *T*2 magnetic resonance imaging showed an abnormality from the lateral aspect of the right sphenoid sinus extending along the right superior orbital fissure.

A computed tomography (CT) scan showed the homogenous complete opacification of the bilateral sphenoid and frontal sinuses. Erosion was seen within the anterior part of the right lateral sphenoid sinus wall, which was closely related to the superior orbital fissure. There was no skull base defect or dehiscence (Figure 1). A magnetic resonance imaging (MRI) scan showed mucosal thickening in all the paranasal sinuses, with increased central *T*1 signal intensity and decreased signal intensity on FLAIR and *T*2-weighted images. A focal extension of signal abnormality was noted from the lateral aspect of the right sphenoid sinus, extending along the right superior orbital fissure, which was associated with mild dural thickening and enhancement in the anteromedial aspect of the right middle cranial fossa. The extension of dural thickening and enhancement was also noted along the orbital apex surrounding the optic nerve (Figure 2).

### 2.2. Management and Surgery

**Figure 3:**
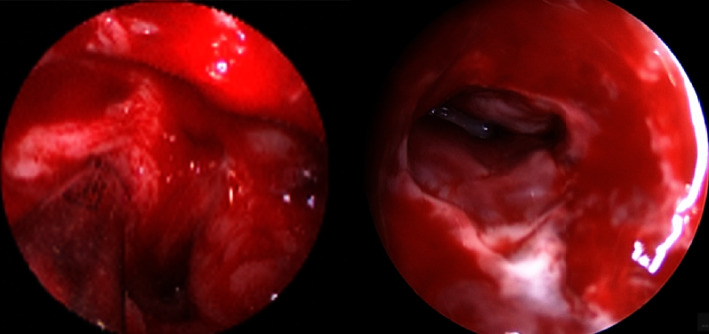
An intraoperative endoscopic view of adhesion in the right opticocarotid recess (left) and the view after the release of the adhesions and the evacuation of the pus (right).

The patient was given intravenous dexamethasone, clindamycin, and ceftriaxone for a duration of three days. On the fifth day of presentation, he underwent a revision bilateral endoscopic FESS, which revealed stenotic scarring on both maxillary antrostomies, with thick hydrated mucin and a polypoid fibrotic mucosa in the maxillary sinus, as well as multiple small pyoceles in the maxillary sinuses. Also, in the ethmoid cavity, the mucosa was polypoidal bilaterally and filled with small pyoceles. Then, trans-ethmoidal sphenoidotomy revealed scar tissues sealing over right opticocarotid recess (OCR; Figure 3). It was opened, and the retained pus was suctioned to evacuate the retained collection. Dehiscence over the carotid and optic nerves was also observed.

### 2.3. Postoperative Management and Outcomes

The patient was discharged on the second day after the surgery, with close follow-up. His diplopia began to improve on the third day and then resolved completely on the eighth day of follow-up. He continued receiving oral antibiotics, a fluticasone-salmeterol inhaler, and oral prednisone at 40 mg for 14 days, as well as normal saline irrigation and mometasone nasal spray postoperatively. On ophthalmological follow-up 2 months after surgery, the patient showed a full range of movement. The endonasal scope revealed healthy-looking mucosa, including OCR. The histopathology study of the surgical specimen demonstrated mucus and inflammatory cells, and a bacterial stain and culture were negative, as was a fungal culture. The patient has been followed up for the next 5 years, with no recurrence.

## 3. Discussion

The sphenoid sinus is adjacent to critical structures, such as the middle cranial fossa, cavernous sinus, clivus and pons, cribriform plate, and posterior nasal cavity. It is separated from the surrounding structures by a wall barrier that consists of either thick bone, thin bone, or only mucosal membrane [7]. A defect in the sphenoid sinus wall, which can be congenital or acquired, can lead to the extension of diseases to the surrounding cranial nerves and, thus, unfavorable outcomes [8]. In particular, for the sphenoid sinus, the pattern of pneumatization may expand in different directions, with various extensions to adjacent structures, such as the optic nerve, cavernous sinus, the internal carotid artery, the frontal lobe, the ventral surface of the brainstem, cranial nerves III–VI, and the pituitary gland [9]. One study found that the most common type of pneumatization is the lateral wall extension of the sphenoid sinus (29.5%), while the lesser wing type, which includes superior wall, OCR, and tuberculum recesses, was observed in only 7% of cases [10]. Considering this, pneumatization can lead to a direct increase in sphenoid sinus pathology and extension to the surrounding structures. In our case, this presentation occurred following ABRS due to either the direct extension of the inflammatory process from the inflamed sinuses or the dehiscence of the abducens nerve in the OCR.

The course of ABRS is typically benign and self-limiting, and it can be managed with self-care measures such as analgesia, decongestants, and normal saline rinses. Oral antibiotics are recommended if symptoms do not resolve after 10 days or the severity of symptoms increases by the fifth day [1]. Complications are rare and include orbital extension, which is thought to occur either directly through osteitis of the lamina papyracea or in a retrograde venous pathway via thrombophlebitis of the communicating veins [11, 12]. The intracranial complications of ABRS exhibit a similar pathophysiology: the direct extension of osteitis from the frontal sinus, the extension of osteitis through a congenital or trauma-induced defect in the skull base, or retrograde thrombophlebitis of the diploic veins extending to the dura [13]. The presence of complications may encourage surgical intervention, specifically the drainage of the sinuses to relieve pressure, rapidly remove the inflammatory triggers, and isolate the organism and thus allow culture-specific antimicrobial treatment.

If a patient presents with atypical symptoms of acute rhinosinusitis that indicate an extension beyond the sinuses, such as diplopia, stroke, or vision loss, this suggests a more aggressive disease, such as invasive fungal sinusitis. The management of invasive fungal sinusitis requires systemic antifungal treatment, urgent surgical debridement of the necrotic tissue, and the reversal of the patient's immune status [1].

In patients with CRS, synechiae formation can be a sequel to the stripping of the mucosa during FESS. This complication is associated with more severe types of CRS and multiple surgeries. During the healing of the mucosa after surgery, neoosteogenesis, mucoceles, and adhesions may develop [14]. The presence of synechiae, as in our case, can alter sinus drainage, resulting in an encouraging habitat for bacteria and increasing the occurrence and severity of infections [15, 16]. Therefore, in modern FESS, mucosa-preserving surgery is advised to avoid such complications and outcomes [15].

The pathophysiology of the acute exacerbation of CRS (AECRS) is believed to be triggered by a viral infection, most likely a rhinovirus, which, in turn, tends to increase the abundance levels of other microbial pathogens, such as bacteria. This infection activates the host inflammatory pathway and promotes the classical manifestations of AECRS [1]. In addition, the microbiological profile for AECRS is different from that of classical ABRS, and culture-guided therapy is recommended [17]. However, only around 50% of patients suspected of having ABRS yield a positive culture [4]. In our patient, the culture results were negative, which can be explained by the patient receiving antibiotics prior to the surgery. Some studies have identified asthma, CRS without nasal polyposis, sinonasal outcome test scores greater than 24, a history of sinus surgery, a high body mass index, hay fever, and migraine as risk factors for AECRS [18, 19].

The most important prognostic factor for abducens nerve palsy depends on the underlying type of pathology and the extent of the damage to the nerve and blood supply. Destructive lesions involving the sinus wall and blood supply, such as invasive fungal sinusitis and invasive neoplasms, have been associated with poor recovery, while compressive masses without invasion have been associated with optimal recovery within two weeks [6]. For abducens nerve palsy secondary to ABRS, the literature shows partial recovery in only three cases and no resolution in only one case of 24 [6, 20, 21] (Table 1). The average duration of resolution for neuropraxia was 38.3 days (Table 1). Early intervention was found to be the most important factor in a favorable outcome and complete recovery, as delayed, proper intervention was associated with residual nerve damage [39].

## 4. Conclusion

Acute bacterial rhinosinusitis can present with atypical symptoms and lead to serious complications, such as abducens nerve palsy. Early diagnosis, appropriate management, and timely referral to a multidisciplinary team are crucial in preventing residual nerve damage and ensuring favorable outcomes.

## Figures and Tables

**Table 1:** Summary of cases of abducent nerve palsy secondary to sinus pathology in the literature.

Study	Etiology	Presentation	Laterality	Clinical grading system^†^	Management	Prognosis	Time to resolution
Miller et al. [6]	AFRS	Diplopia	Bilateral	Grade 2	Surgery	Complete recovery	2 weeks
	AFRS	Diplopia	Right	Grade 2	Surgery	Complete recovery	2 weeks
	AFRS	Diplopia	Left	Grade 2	Surgery, steroids, and oral antibiotics	Partial recovery	1 month
	ABRS	Diplopia	Right	Grade 2	Surgery and intravenous antibiotics	Partial recovery	34 days

Gupta et al. [22]	ABRS	Diplopia, headache and fever	Left	Grade 2	Surgery and intravenous antibiotics	Complete recovery	2 days

Kordrostami and Parmar [23]	ABRS	Diplopia and visual loss	Right	Grade 4	Surgery and intravenous antibiotics	Full improvement of visual acuity with preset of sixth-nerve palsy	2 months

Mograbi and Soudry [24]	ABRS	Diplopia	Right	Grade 2	Surgery, steroids, and oral antibiotics	Complete recovery	1 month
	CRS	Diplopia	Left	Grade 2	Surgery and antibiotics	Complete recovery	2 weeks

Ada et al. [25]	ABRS	Diplopia and headache	Right	Grade 2	Antibiotics and steroids	Complete recovery	2 weeks

Lee et al. [20]	Chronic invasive fungal sinusitis	Diplopia	Bilateral	Grade 2	Surgery and antifungal treatment	Complete recovery	17 months
	Sphenoid sinus fibrosis	Diplopia	Left	Grade 2	Surgery and antibiotics	No resolution	No resolution
	Fungal ball	Diplopia and headache	Left	Grade 2	Surgery	Complete recovery	1 month
	ABRS	Retroorbital pain and progressive diplopia	Left	Grade 2	Surgery and antibiotics	Complete recovery	4 months

Fockaert et al. [26]	AFRS	Diplopia and retroorbital pain	Left	Grade 2	Surgery	Complete recovery	1 month

Siu et al. [27]	ABRS	Diplopia and headache	Right	Grade 2	Surgery and antibiotics	Complete recovery	6 weeks

Safouris et al. [28]	ABRS	Diplopia and headache	Left	Grade 2	Antibiotics and steroids	Complete recovery	2 weeks

Del Brutto and Caputi [29]	ABRS	Diplopia and headache	Left	Grade 2	Antibiotics	Complete recovery	3 weeks

Mubaidin and Hairi [7]	ABRS	Fever, diplopia and headache	Bilateral	Grade 2	Antibiotics	Complete recovery	4 weeks

Harugop et al. [21]	Fungal ball	Fever, diplopia and headache	Left	Grade 2	Surgery, intravenous antibiotics, and local anti-fungal treatment	Complete recovery	12 weeks
	NR	Decrease vision, fever and diplopia	Right	Grade 2	Surgery and anti-fungal treatment	Partial recovery	12 weeks

Trinidade et al. [30]	Fungal ball	Diplopia	Right	Grade 2	Surgery	Complete recovery	3 weeks

Ceylan et al. [31]	CRS	Diplopia and headache	Bilateral	Grade 2	Surgery, steroids, and oral antibiotics	Complete recovery	6 weeks

Lapusneanu et al. [32]	ABRS	Diplopia and headache	Right	Grade 2	Surgery, steroids, and oral antibiotics	Complete recovery	1 month

Giordano et al. [33]	ABRS	Diplopia, ptosis and headache	Left	Grade 2	Surgery and intravenous antibiotics	Complete recovery	4 days

Vazirnezami et al. [34]	AFRS	Diplopia and headache	Right	Grade 2	Surgery	Complete recovery	5 weeks

Muneer and Jones [35]	Mucocele	Diplopia and headache	Right	Grade 2	Surgery, steroids, and oral antibiotics	Complete recovery	2 days
	Mucocele	Diplopia and periorbital cellulitis	Right	Grade 2	Surgery and steroids	Complete recovery	2 days
	Mucocele	Diplopia	Right	Grade 2	Surgery	Complete recovery	1 day

Geçirilmesi [36]	Mucocele	Diplopia	Right	Grade 2	Surgery	Partial recovery	6 months

Hill et al. [37]	Mucocele	Diplopia, headache and blurred vision,	Right	Grade 4	Surgery and antibiotics	The third nerve palsy had resolved. There was no change in his visual acuity however	3 months
	Mucocele	Ptosis, diplopia and headache	Left	Grade 2	Surgery	Minimal residual third nerve weakness	1 year

AFRS: allergic fungal rhinosinusitis, ABRS: acute bacterial rhinosinusitis. †: clinical grading system is adapted from Al Anazy and Al-Dousary's classification system [38].

## Data Availability

The case report and literature review data used to support the findings of this study are included within the article.
